# A Calcium Enterolith in a Patient with Crohn's Disease and Its In Vitro Dissolubility in Citric Acid

**DOI:** 10.1155/2017/2951547

**Published:** 2017-09-07

**Authors:** Masaya Iwamuro, Haruo Urata, Sakiko Hiraoka, Masayasu Ohmori, Yoshitaka Kondo, Yoshiro Kawahara, Hiroyuki Okada

**Affiliations:** ^1^Department of Gastroenterology and Hepatology, Okayama University Graduate School of Medicine, Dentistry, and Pharmaceutical Sciences, 2-5-1 Shikata-cho, Kita-ku, Okayama 700-8558, Japan; ^2^Department of General Medicine, Okayama University Graduate School of Medicine, Dentistry, and Pharmaceutical Sciences, 2-5-1 Shikata-cho, Kita-ku, Okayama 700-8558, Japan; ^3^Central Research Laboratory, Okayama University Medical School, 2-5-1 Shikata-cho, Kita-ku, Okayama 700-8558, Japan; ^4^Department of Gastroenterological Surgery, Okayama University Graduate School of Medicine, Dentistry, and Pharmaceutical Sciences, 2-5-1 Shikata-cho, Kita-ku, Okayama 700-8558, Japan; ^5^Department of Endoscopy, Okayama University Hospital, 2-5-1 Shikata-cho, Kita-ku, Okayama 700-8558, Japan

## Abstract

The microstructure and dissolubility of a calcified enterolith and enterolith pieces removed from a 26-year-old Japanese woman with Crohn's disease were analyzed using scanning electron microscopy and energy dispersive X-ray spectroscopy. The enterolith showed a multilayered structure with fatty acid calcium and magnesium phosphate. The amount of calcium, magnesium, and phosphate decreased after they were immersed in a citric acid solution, suggesting a potential contribution of acidic aqueous solution to elute inorganic substances contained in calcified enteroliths. This is the first study to investigate the in vitro dissolubility of calcified enteroliths induced by citric acid solution.

## 1. Introduction

An enterolith is a mass found in the small or large intestine. False enteroliths are formed from indigestible substances trapped in the intestinal tract such as bezoars, gallstones, or foreign objects, whereas true enteroliths are formed from the precipitation of enteric contents [1, 2]. Formation of true enteroliths is generally caused by bowel stasis due to strictures or diverticula, followed by precipitation of inorganic substances or bile acids. Such precipitation of materials contained in intestinal juice is thought to be facilitated by an acid-base imbalance and bacterial overgrowth [3–8]. Most cases with enteroliths were asymptomatic and did not require specific treatment. In symptomatic cases with impacted enteroliths causing ileus or inflammation, endoscopic treatment such as dilation for the stricture of the bowel and fragmentation will be attempted in some cases [9]. If the enterolith is inaccessible via endoscopy devices or refractory for endoscopic treatment, surgical treatment is generally inevitable since enteroliths are unresponsive to medical treatment and successful chemical dissolution of enteroliths has never been reported.

Recently, we removed a calcified enterolith from a patient with Crohn's disease. This paper will discuss how we performed two basic studies to reveal the microstructure and dissolubility of the calcified enterolith by using citric acid solution.

## 2. Materials and Methods

### 2.1. Patient History

**Figure 1:**
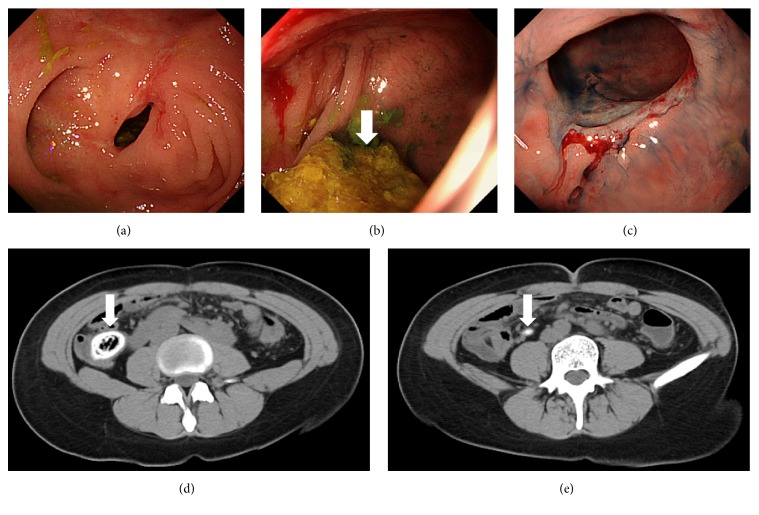
Endoscopic and radiologic images. Colonoscopy performed 15 months previously shows ulceration and stenosis (a) and a mass trapped in the semi-closed lumen in the cecum ((b), arrow). Another ulceration is observed in the anastomotic site (c). CT shows a calcified mass in the cecum ((d), arrow) and fecalomas within the appendix ((e), arrow).

A 26-year-old Japanese woman presented with abdominal pain, fever, and watery diarrhea. The patient was diagnosed as having Turner syndrome at the age of six years and had been treated for Crohn's disease since the age of 18 years. She underwent left hemicolectomy at the age of 20 years due to complete obstruction of the descending colon. Adalimumab was administered previously, but it was discontinued due to hair loss as a side effect of the drug. The patient had been treated with 10 mg/day of prednisone on admission. A colonoscopy performed 15 months prior to her admission revealed ulceration and stenosis of the cecum (Figure 1(a)). A mass that appeared to be feces was observed in the semi-closed lumen in the cecum (Figure 1(b)). Ulceration was also observed in the anastomotic site (Figure 1(c)). She had a bowel movement once every day. On admission, computed tomography scanning demonstrated a calcified mass that was impacted in the cecum (Figure 1(d)) and fecalomas within the appendix (Figure 1(e)). Laboratory testing revealed slightly elevated levels of C-reactive protein (0.36 mg/dL), whereas the white blood cell count was within the normal ranges (7070/*μ*L). An ileocecal resection was performed under the diagnosis of impacted cecal enterolith. Although appendiceal wall thickening was not noted, infiltration of neutrophils within the mucosa and lymphocytes and eosinophils within the subserosa and adipose tissue were confirmed microscopically. Thus, the final diagnosis of an enterolith impacted in the cecum and acute appendicitis was made. Her abdominal pain, fever, and diarrhea disappeared after the surgery. Since inflammation was pathologically noted in the appendix, we speculate that the impacted cecal enterolith induced appendicitis.

### 2.2. Structure and Dissolubility Analysis of an Enterolith

**Figure 2:**
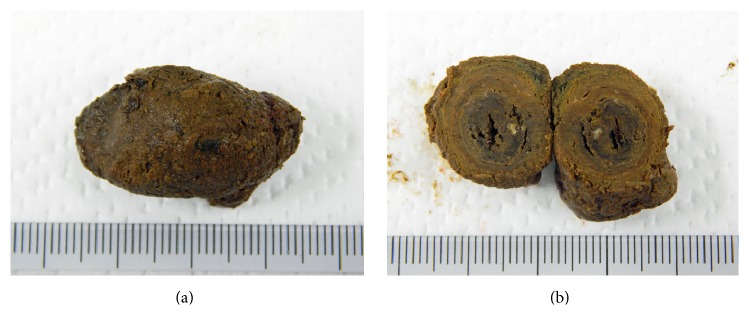
Photographs of the enterolith. Enterolith of 3 × 2 cm in size was surgically removed (a). The cut surface shows a multilayered structure (b).

**Figure 3:**
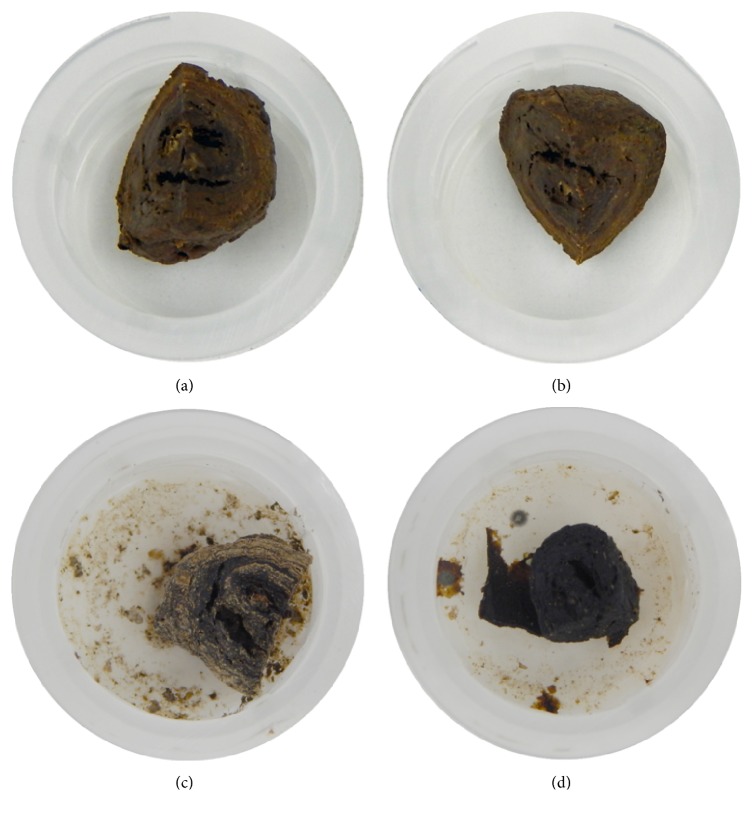
Photographs of the enterolith pieces. A piece before immersing in double distilled water (a) and a piece before immersing in 2% citric acid solution (b). After 120 hours of immersion and 30 minutes of air-drying, the color of the enterolith piece immersed in citric acid solution turned to black and appeared to have shrunken (d), compared with the piece immersed in water (c).

Figure 2 shows the surgically removed enterolith of 3 × 2 cm in size (Figure 2(a)). Multiple-layered structures were seen in the cut surface, resembling annual rings of a tree (Figure 2(b)). The enterolith was cut into four pieces, and one piece was used in an infrared spectroscopy analysis. Infrared spectroscopy revealed that the enterolith was mainly composed of two components: fatty acid calcium and magnesium phosphate. Two enterolith pieces were placed in cell strainers with 40 *μ*m pores (Corning Inc., Corning, NY, USA). The cell strainers were set in 50 mL conical polystyrene tubes. A citric acid solution was prepared by dissolving citric acid powder for food additives (Kenei Pharmaceutical Co., Osaka, Japan) in double-distilled water. One piece (Figure 3(a)) was completely immersed in double-distilled water, and the other (Figure 3(b)) was immersed in a 2% citric acid solution. After 120 hours of immersion at room temperature (25°C) without stirring, the enterolith pieces were removed from the cell strainers and air dried for 30 minutes. The color of the enterolith piece immersed in citric acid solution turned black in color and appeared to have shrunken (Figure 3(d)), compared with the piece immersed in water (Figure 3(c)). The percentage of lysis was calculated by comparing the weight of preimmersion with that of postimmersion. The weight was 1.717 g at preimmersion and 0.842 g at postimmersion in double-distilled water, indicating a 51.0% decrease in weight. Meanwhile, the weight changed from 1.330 g to 0.440 g after immersing in 2% citric acid solution, indicating a 66.9% decrease in weight.

**Figure 4:**
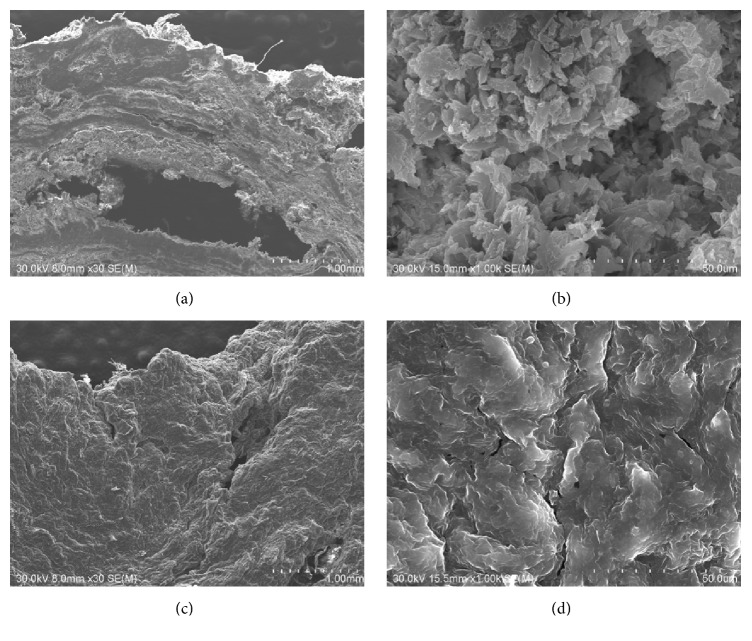
Scanning electron microscopy images. The enterolith piece immersed in water revealed a multilayered structure (a), containing crystalloid microparticles (b). In the enterolith piece immersed in citric acid solution, crystalloid microparticles were absent (c, d).

**Figure 5:**
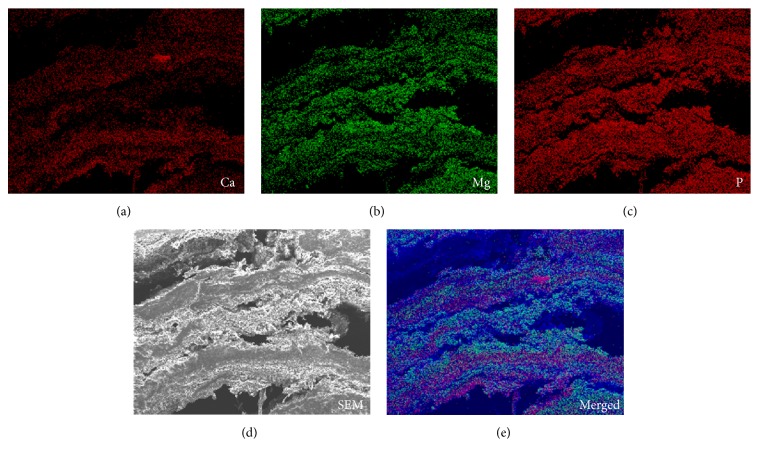
Elemental mapping images. The enterolith piece immersed in water contained calcium (a), magnesium (b), and phosphate (c). A photograph merged with a scanning electron microscopy image (d) and mapping images of calcium and magnesium showed that the enterolith piece was composed of two kinds of layers (e).

**Figure 6:**
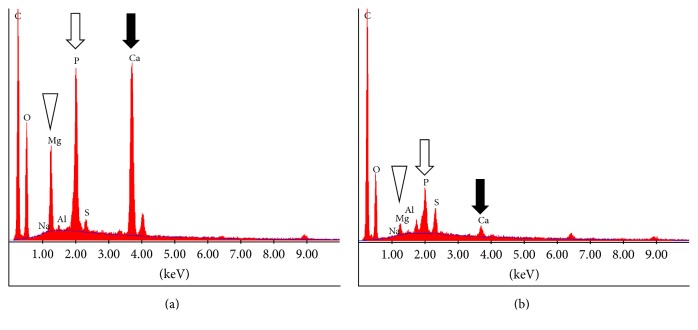
Spectra obtained using energy-dispersive X-ray spectroscopy. Calcium, magnesium, and phosphate exist in the enterolith piece immersed in double-distilled water (a). The amount of these elements was decreased in the piece immersed in the citric acid solution (b).

Subsequently, we investigated the microstructure and elemental composition of the enterolith samples immersed in water and of those in the citric acid solution. We used S4800 scanning electron microscopy (Hitachi, Tokyo, Japan) and an EDAX Genesis APEX2 system (Ametek, Paoli, PA, USA). Elemental mapping of iron was also performed with S4800 scanning electron microscopy and EDAX Genesis APEX2 energy-dispersive X-ray spectroscopy. Scanning electron microscopy analysis of the enterolith piece immersed in water revealed a multilayered structure (Figure 4(a)), which was partly composed of crystalloid microparticles (Figure 4(b)). In the enterolith piece immersed in citric acid solution, crystalloid microparticles were absent (Figures 4(c) and 4(d)). Elemental mapping revealed that the enterolith piece immersed in water contained calcium (Figure 5(a)), magnesium (Figure 5(b)), and phosphate (Figure 5(c)). It was noteworthy that the magnesium and phosphate were deposited in approximately the same areas, whereas the calcium was deposited in different areas from magnesium and phosphate. A photograph merged with scanning electron microscopy image (Figure 5(d)) and mapping images of calcium and magnesium clearly showed that the enterolith piece was composed of two kinds of layers (Figure 5(e)). Spectra obtained with energy-dispersive X-ray spectroscopy showed that calcium, magnesium, and phosphate existed in the enterolith piece immersed in water (Figure 6(a)), whereas the amount of these elements was decreased in the piece immersed in citric acid solution (Figure 6(b)).

## 3. Discussion

This report focused on the microstructure and dissolution of an enterolith removed from a patient with Crohn's disease. True enteroliths are mainly subdivided into two types: bile acid enteroliths and calcified enteroliths [10]. Primary bile acids (i.e., cholic acid and chenodeoxycholic acid) are metabolized into secondary bile acids (i.e., deoxycholic acid and lithocholic acid) by anaerobic bacteria. These bile acids are relatively insoluble in the acidic milieu, leading to the formation of bile acid enteroliths in the proximal small bowel [7, 11–13]. Meanwhile, calcified enteroliths are reportedly composed of calcium phosphate, calcium oxalate, and calcium carbonate. These salts are insoluble in alkaline environments and thus can precipitate and form calcified enteroliths most often in the terminal ileum [4, 8, 10, 14]. As described previously, precipitation of substances is generally induced by bowel stasis followed by acid-base imbalance and bacterial overgrowth. Diverticular disease of the bowel is the most common etiology to lead enterolith formation, followed by stricture and narrowing of the intestine because of tuberculosis or Crohn's disease and postsurgical alteration of the alimentary tract [10]. The presented patient had a calcified enterolith in the cecum where stricture existed due to Crohn's disease. These features are in concordance with findings reported in earlier reports.

In the present patient, the enterolith showed a multiple-layered structure, resembling annual rings of a tree (Figure 2). Infrared spectroscopy analysis showed that the enterolith was mainly composed of fatty acid calcium and magnesium phosphate. Moreover, scanning electron microscopy observation of the enterolith piece immersed in water revealed that calcium-containing layers and magnesium phosphate-containing layers constitute the multilayered structure of the enterolith (Figure 5). Consequently, we speculate that the enterolith gradually increased diameter because of deposition of fatty acid calcium and magnesium phosphate in an alternating manner.

To our knowledge, this report is the first to investigate the in vitro dissolubility of calcified enteroliths induced by citric acid solution. The weight of the enterolith piece immersed in double-distilled water for 120 h showed a 51.0% decrease. We speculate that evaporation of water from the enterolith by air-drying is the main cause of the weight change, although some amount of substances might have been liquated from the cut surface into water. Meanwhile, the enterolith piece immersed in the 2% citric acid solution showed a 66.9% decrease in weight. Energy-dispersive X-ray spectroscopy revealed that the amount of calcium, magnesium, and phosphate was lower in the enterolith piece immersed in the citric acid solution, compared with that in water. These results suggest that calcium, magnesium, and phosphate were eluted by the citric acid solution. Moreover, based on the results obtained using energy-dispersive X-ray spectroscopy, we consider that crystalloid microparticles in the enterolith piece immersed in water (Figure 4(b)) were inorganic substances such as calcium, magnesium, and phosphate.

As described above, calcified enteroliths are considered to be formed in alkaline environments. An in vivo equine study revealed that horses with enteroliths had higher calcium, magnesium, phosphorus, and sulfur concentrations and higher pH in colonic contents than control horses [15, 16]. Conversely, calcified enteroliths may partly dissolve in lower pH, as shown in the present study. We also speculate that the residue that remained after immersion in the citric acid solution (Figure 3(d)) was organic substances, since the enterolith piece contained fatty acid and a higher amount of carbon was detected (Figure 6(b)).

This study has several limitations. First, because of the small size of the removed enterolith, only two pieces were used in the dissolution experiment. Therefore, statistical analysis was inapplicable to compare weight changes between the enterolith piece immersed in double-distilled water and that in citric acid solution. Second, the composition of calcified enteroliths is probably diverse in each patient [1, 17], so an acidic aqueous solution may not be useful in dissolving all calcified enteroliths. Lastly, because we examined dissolubility of enteroliths in vitro, further study is required prior to the introduction of a citric acid solution into clinical practice. For example, although a citric acid solution can be ingested in patients without intestinal obstruction, the degree of acidity or alkalinity will be altered under the influence of digestive juices such as gastric acid, bile acid, and a mucus-rich, bicarbonate-containing, alkaline juice produced by Brunner's glands. A possible approach is infusing acidic solution into the diseased bowel via an ileus tube or endoscopy. Investigations with regard to the utility and safety of a citric acid solution for the treatment of enteroliths in clinical settings are required as well.

In summary, we investigated the microstructure of the enterolith removed from the patient with Crohn's disease. This study revealed that inorganic substances constituting the enterolith were eluted and the weight of the enterolith piece was decreased after immersing it in a citric acid solution in vitro. We believe that an acidic aqueous solution has potential for the treatment of enteroliths.
